# Correction

**DOI:** 10.1080/20018525.2026.2678116

**Published:** 2026-05-24

**Article title**: Multidisciplinary and stratified non-pharmacological airway clearance intervention to prevent stroke-associated pneumonia: a quasi-experimental study

**Authors**: Yingchun Chen, Lihong Huang, Xi Huang, Inhye Park, Chao Wu, Yueli Gong, Jie Du, Jiawen You and Yang Liu

**Journal**: European Clinical Respiratory Journal

**Bibliometrics**: Volume 13, Number 01, pages 1-18

**DOI**: https://doi.org/10.1080/20018525.2026.2661516

The authors requested an update to the Author Contributions section. The updated version is provided below. This correction does not affect the description, interpretation, or original conclusions of the article. The authors apologize for any inconvenience caused.

**Author contributions**

CRediT: **Yingchun Chen**: Data curation, Formal analysis, Investigation, Methodology, Project administration, Resources, Supervision, Writing – original draft, Writing – review & editing, Funding acquisition; **Lihong Huang**: Data curation, Formal analysis, Methodology, Writing – original draft, Writing – review & editing; Xi Huang: Data curation, Formal analysis, Writing – review & editing; **In Hye Park**: Methodology, Software, Writing – review & editing; Chao Wu: Conceptualization, Resources, Supervision, Validation, Visualization; **Yueli Gong**: Conceptualization, Resources, Software, Supervision, Validation, Visualization; **Jie Du**: Investigation, Resources, Software, Supervision, Validation, Visualization; **Jiawen You**: Data curation, Formal analysis, Methodology, Writing – review & editing; **Yang Liu**: Data curation, Formal analysis, Methodology, Writing – original draft, Writing – review & editing,Funding acquisition.

